# Multifactorial Risk Profiling of Overuse Injuries in Elite High School Basketball Players in Japan: A Cluster Analysis Approach

**DOI:** 10.7759/cureus.85134

**Published:** 2025-05-31

**Authors:** Yuh Yamashita, Nozimi Hamachi, Nobuya Harada, Hiroyuki Konishi, Norihisa Matsumoto, Takeyoshi Shimoda, Haruki Kogo

**Affiliations:** 1 Department of Rehabilitation, Wakayama Professional University of Rehabilitation, Wakayama, JPN; 2 Faculty of Rehabilitation Sciences, Reiwa Health Sciences University, Fukuoka, JPN; 3 Department of Physical Therapy, School of Health Sciences at Fukuoka, International University of Health and Welfare, Fukuoka, JPN

**Keywords:** basketball, cluster analysis, high school athletes, overuse injury, physical capacity, training exposure

## Abstract

Objective

This study aimed to comprehensively evaluate multiple factors, including body composition, physical function, and training characteristics, in high school basketball players to identify the risk factors associated with overuse injuries, develop risk profiles using cluster analysis, and examine the relationship between these profiles and injury occurrence.

Materials and methods

Eighty male high school basketball players participated in this study. Data were collected on physical function, including grip strength, vertical jump, side-step test, and quadriceps muscle thickness; body composition, including skeletal muscle mass, total body water, and body fat percentage; training characteristics; and demographic variables. Overuse injury was defined as chronic pain or discomfort caused by sports activities within the past six months, diagnosed by a medical professional, and resulting in limitations in sports participation. Risk factors were identified using univariate analysis. A hierarchical cluster analysis was conducted to classify players into distinct risk profiles based on the extracted variables. The association between these cluster profiles and the incidence of overuse injuries was examined using the chi-square test and logistic regression analysis. Predictive accuracy was further assessed using receiver operating characteristic (ROC) curve analysis.

Results

Univariate analysis identified daily training duration (p = 0.034), vertical jump performance, and skeletal muscle mass index (p = 0.071) as factors associated with overuse injuries. Based on these variables, the cluster analysis categorized players into four distinct risk profiles. Cluster 3, characterized by low physical function and longer training hours, demonstrated the highest incidence of overuse injuries (56.2%). Logistic regression analysis revealed a significantly elevated injury risk in cluster 3 (OR = 25.7; 95% CI: 2.7-241.1; p = 0.005). ROC analysis showed good predictive performance of the model (AUC = 0.77).

Conclusions

Imbalances between physical function and training load are key contributors to overuse injuries in high school basketball players. Risk profiling using cluster analysis is a practical and effective method for identifying individuals at risk and may facilitate the development of personalized injury prevention strategies for athletes.

## Introduction

Overuse injuries, unlike acute injuries, do not result from a single identifiable traumatic event. Instead, they develop gradually as a consequence of repeated microtrauma associated with cumulative loading on tissues [1]. Basketball, on the other hand, is a high-intensity sport that involves repetitive movements, including jumping, rapid directional changes, short sprints, and abrupt stops. These repetitive actions place athletes at considerable risk of overuse injuries. In particular, high school athletes have elevated risk due to the overlap of skeletal immaturity and increasing training loads during growth spurts, compounded by changes in physical function and body composition [2]. Overuse injuries are a common concern among athletes, with a prevalence of approximately 30% [3]. In Japan, a retrospective study involving school-aged children and adolescents reported that the prevalence of overuse injuries increases with age, reaching 25% among high school students [4]. Once developed, these injuries often become chronic and require prolonged recovery, leading to a decline in athletic performance, extended periods away from competition, and potentially shortened athletic careers [2,5].

Several key risk factors for overuse injuries have been identified, including excessive cumulative training loads [1,5], insufficient recovery time [5], inadequate muscle strength and flexibility [6], and imbalances in acute-to-chronic workload ratios [7]. These findings underscore the importance of quantitatively monitoring training loads and the need for individualized load management strategies tailored to each athlete [8]. In addition, aspects of physical function, such as muscular strength and jump performance, have been recognized as potential predictors of injury risk through mechanisms, including localized load accumulation and compromised motor control [9]. Athletes with insufficient training backgrounds are particularly vulnerable to injuries following sudden increases in training intensity or volume [10]. Importantly, these risk factors rarely act in isolation; rather, injuries often emerge through complex interactions among physical, psychological, and environmental variables [10,11].

Despite the multifactorial nature of overuse injury risk, the existing literature has focused mainly on isolated variables, which may limit the effectiveness of prevention strategies [9,10]. Therefore, there is a growing need for integrative approaches that consider multiple interacting factors to enable the development of individualized risk profiles [12]. Recent advances in clustering techniques and machine learning have enabled the classification and visualization of complex multivariate risk patterns, offering new possibilities for personalized injury prevention strategies [13,14]. However, few studies have comprehensively analyzed a broad range of relevant factors, including body composition, physical function, and training characteristics, in an integrated manner to visualize individual risk profiles. Moreover, few studies have examined the relationship between data-driven profiles and actual injury occurrences using clustering techniques. Therefore, the development of practical athlete classification models applicable in real-world settings remains a significant challenge.

In Japan, elite-level high school basketball teams are especially competitive, with athletes engaging in rigorous year-round training programs and frequent tournaments. These conditions may increase players’ vulnerability to overuse injuries, particularly during adolescence when their musculoskeletal systems are still developing [15]. The current study aimed to comprehensively evaluate multiple domains, that is, body composition, physical function, and training status, in high school basketball players. Specifically, we aimed to identify the risk factors associated with overuse injuries, construct risk profiles using cluster analysis, and investigate the relationship between these profiles and actual injury incidence. This study contributes to the development of a practical and reproducible screening tool to support injury prevention efforts in athletic populations.

## Materials and methods

Participants

This study included 91 male high school basketball players from F High School’s team. The team has consistently competed at the top national level in Japan for many years, participating in national tournaments and maintaining a high level of performance. The inclusion criteria were team enrollment at the time of the survey and current or past participation in training or competitions. The participant pool included players with no history of injuries; those who sustained traumatic injuries such as sprains, contusions, or fractures due to contact or falls during games or practice; and those who had been diagnosed with or exhibited symptoms suggestive of overuse injuries by a medical professional. The diagnosed conditions included Osgood-Schlatter disease, jumper’s knee, shin splints, stress-related periostitis, and lumbar spondylolysis.

Exclusion criteria were (1) players in the acute phase of injury (within two weeks of onset), (2) those with pain or functional impairments that interfered with testing, (3) players with a history of surgery, and (4) injuries sustained outside of basketball-related activities.

In this study, overuse injury was defined as “an injury caused by the accumulation of repetitive microtrauma without a single identifiable inciting event.” This definition is based on the consensus statement by the International Olympic Committee, which is widely recognized in the context of load management and injury prevention in sports [4]. To ensure consistency in the classification and facilitate comparisons with other studies, we adopted the categorization framework proposed by Orchard et al. [16]. Overuse injury data were collected using a structured self-report questionnaire administered at the time of the physical assessment. Participants were asked to report any symptoms, diagnoses, or medical consultations related to conditions of overuse injuries they had experienced within the past six months.

After applying the inclusion and exclusion criteria, 80 players were included in the study. A total of 11 players were excluded: three were in the acute phase of injury (within two weeks of onset), four were currently injured but not in the acute phase and were unable to participate in testing, two experienced pain that interfered with testing, and two had missing data.

The study was conducted in accordance with the principles of the Declaration of Helsinki. The study protocol, including its objectives and procedures, was explained verbally and in writing to the school principal, team coaches, participating athletes, and their legal guardians. Participation was voluntary, and those who declined to participate or withdrew from the study at any point did not face any disadvantages. Written informed consent was obtained from all the participants and their guardians. All personal information was anonymized, and the collected data were used solely for research purposes, with careful attention to privacy and confidentiality. This study was reviewed and approved by the Institutional Review Board of Reiwa Health Sciences University (approval number: 24-008).

Measures

Demographic and Training Characteristics

Participants reported their age, dominant hand and foot, years of basketball experience, and playing position (center, forward, or guard). Additionally, information was collected on daily training duration (hours and minutes), total weekly training time (hours), and training frequency (days per week).

To assess dietary awareness, participants answered two yes/no questions: (1) Do you take care to eat properly before training? (2) Do you adjust your diet based on your training volume?

Physical Assessments

Physical assessments were conducted by 16 trained examiners at the F High School gymnasium. They included the following eight items: height, body weight, body composition, grip strength, quadriceps muscle thickness, seated forward bend, side-to-side jump, and vertical jump. To minimize measurement variability, each examiner was assigned specific test items, and a standardized pre-study training session was conducted to ensure consistency in measurement procedures among all assessors.

Height and Body Weight

Participants were barefoot during measurements. Height was measured using a stadiometer (SECA213; SECA Japan), and body weight was measured using a body composition analyzer (InBody470; InBody Japan).

Body Composition

Body composition was assessed using the InBody470 analyzer, which provided the following indices: total body water, protein mass, mineral mass, body fat mass, fat-free mass, body mass index (BMI), percent body fat, skeletal muscle mass, resting energy expenditure, bone mineral content, and skeletal muscle mass index (SMI).

Grip Strength

Participants stood with their feet shoulder-width apart, arms relaxed at their sides without touching the torso, and forearms in a neutral position. Grip strength was measured using a digital dynamometer (T.K.K.5401; Takei Scientific Instruments), with two alternating trials per hand. The maximum value from each side was recorded, and the average of the two values, normalized by body weight, was used for statistical analysis.

Quadriceps Muscle Thickness

Quadriceps muscle thickness was measured at the midpoint between the anterior superior iliac spine and the superior border of the patella, following the method described by Abe et al. [17]. Measurements were performed using a portable ultrasound device (Viamo SSA-640A; Toshiba Medical Systems) with a 7.5 MHz linear probe (PLT-1204ST; Toshiba Medical Systems) in B-mode. Participants lay supine on a mat in a relaxed state to avoid muscle contractions. The probe was applied transversely without excessive pressure.

The muscle thickness of the rectus femoris was defined as the distance between the subcutaneous fascia and the fascia between the muscles. In contrast, the thickness of the vastus intermedius was defined as the distance from the fascia to the surface of the femur. Each side was measured twice, and the average value was used for analysis. Muscle thickness was normalized to body weight to account for individual differences in growth and physique.

Seated Forward Bend

Flexibility was assessed using the seated forward bending test. Participants sat with their backs and hips against the wall, knees extended, and ankles in a neutral position. A digital sit-and-reach meter (T.K.K.5412; Takei Scientific Instruments) was used. Participants extended both arms forward, placed their second to fifth fingers on the measurement platform, and slid their fingers forward while keeping their thumbs below the platform. The maximum reach of the two trials was used for the analysis.

Side-Step Test

Agility was assessed using the side-step test conducted in the gymnasium [18], in which three parallel lines were marked on the floor: a center line and two outer lines placed 100 cm to either side. Participants stood astride at the centerline. Upon the “start” signal, they performed lateral steps over one outer line, returned to the center, and then crossed the opposite line, repeating the sequence for 20 s. One point was awarded for each line crossed, and the highest score was used for the analysis.

Vertical Jump

Lower limb power was evaluated using a digital vertical jump meter (Jump-MD, T.K.K.5406; Takei Scientific Instruments). Participants wore a waist belt attached to the device and stood at the center of the rubber platform. Before each jump, the pulley system was gently adjusted to eliminate slack in the cord. Participants were instructed to jump as vertically as possible with maximum effort. Two trials were performed, and the maximum value was used for analysis.

Statistical analysis

Statistical analyses were conducted in several stages. Univariate analyses were performed to compare variables between athletes with and without overuse injuries. Independent t-tests were used for continuous variables, and Fisher’s exact test was applied for categorical variables. The significance level for these preliminary comparisons was set at p < 0.1 to allow for a broad exploration of potential explanatory variables in subsequent multivariate analyses [19].

The variables that met this criterion were included in the cluster analysis. Prior to clustering, all variables were standardized using Z-scores. Hierarchical clustering was performed using Ward’s method based on Euclidean distances. To facilitate the visual comparison of physical characteristics across clusters, radar charts were created using Z-scores by employing the matplotlib library in Python.

A one-way analysis of variance (ANOVA) was conducted using the least squares method to examine the differences in the quantitative indicators used for clustering across the identified clusters. The effect size for each ANOVA was calculated as eta squared (η²), indicating the proportion of variance attributable to the clustering factor. When significant main effects were found, post hoc comparisons of least squares means were performed using Student’s t-test. To control for type I errors arising from multiple comparisons, the Bonferroni correction was applied, and the adjusted significance threshold was set according to the number of clusters and comparisons. In addition, Cohen’s d was calculated to assess the magnitude of the pairwise differences between clusters. Interpretation of effect sizes followed Cohen’s (1988) guidelines: for η², values of ≥ 0.01, ≥ 0.06, and ≥ 0.14 were considered small, medium, and large, respectively; for Cohen’s d, thresholds of 0.2, 0.5, and 0.8 indicated small, medium, and large effects, respectively [20].

The association between cluster membership and the occurrence of overuse injuries was further examined using contingency table analysis, and statistical significance was assessed using Fisher’s exact test. Logistic regression analysis was performed to investigate the predictive value of cluster classification for the occurrence of injuries. The cluster group was entered as the independent variable, and the presence or absence of overuse injury was the dependent variable. Odds ratios (OR) and 95% confidence intervals (CI) were determined.

Finally, to evaluate the discriminative performance of the logistic model, a receiver operating characteristic (ROC) curve analysis was conducted, and the area under the curve (AUC) was calculated. All statistical analyses were performed using JMP Pro version 17 (SAS Institute Inc., Cary, NC, USA). The final level of statistical significance was set at p < 0.05.

## Results

Participant characteristics

Table 1 presents the basic characteristics of the study participants. The sample consisted of 80 male high school basketball players with a mean age of 16.5 ± 0.9 years. The mean height and body weight were 174.2 ± 9.3 cm and 65.5 ± 9.4 kg, respectively. The average duration of basketball experience was 8.5 ± 2.4 years. The weekly training volume was 26.0 ± 9.2 h, with a daily training average of 3.8 ± 1.2 h and a training frequency of 6.7 ± 1.1 days per week.

**Table 1:** Demographic, physical, and nutritional characteristics of the participants ^§^ Quadriceps thickness value adjusted for body weight SD: standard deviation, BMI: body mass index, SMI: skeletal muscle mass index

Variables	All participants (n = 80)	
Demographic data; mean ± SD		
Age (years)	16.5	± 0.9
Height (cm)	174.2	± 9.3
Body weight (kg)	65.5	± 9.4
Years of basketball experience (years)	8.5	± 2.4
Total weekly training duration (h)	26.0	± 9.2
Daily training duration (h)	3.8	± 1.1
Weekly training frequency (days/week)	6.7	± 1.2
Player positions; n (%)		
Center	7	9.0
Forward	22	28.0
Guard	52	63.0
Do you take care to eat properly before training?; n (%)		
Yes	22	27.2
No	59	72.8
Do you adjust your diet based on your training volume?; n (%)		
Yes	25	31.3
No	55	68.8
Physical Performance; mean ± SD		
Grip strength (kgf)	41.7	± 7.1
Quadriceps muscle thickness (mm/kg)^§^	40.8	± 5.2
Seated forward bend (cm)	43.7	± 10.1
Side-to-side jumps (cm)	60.9	± 6.1
Vertical jump (cm)	55.2	± 6.1
Body composition parameters; mean ± SD		
Total body water (L)	42.8	± 6.2
Protein (kg)	11.7	± 1.7
Minerals (kg)	3.9	± 0.6
Body fat mass (kg)	7.1	± 2.7
Soft lean mass (kg)	55.2	± 8.0
Fat-free mass (kg)	51.6	± 19.1
BMI (kg/m²)	21.5	± 1.8
Percent body fat (%)	10.7	± 3.5
Skeletal muscle mass (kg)	33.2	± 4.9
Resting energy expenditure (kcal)	1631.4	± 183.2
Bone mineral content (kg)	3.2	± 0.5
SMI (kg/m²)	8.3	± 0.6

In terms of position, there were seven centers (9.0%), 22 forwards (28.0%), and 52 guards (63.0%). Regarding nutritional awareness, 22 participants (27.2%) reported being mindful of their pre-training meals, while 25 (31.3%) indicated that they adjusted their meals according to the training volume.

As for physical function, grip strength averaged 41.7 ± 7.1 kgf, quadriceps muscle thickness adjusted for body weight was 40.8 ± 5.2 mm/kg, sit-and-reach distance was 43.7 ± 10.1 cm, side-to-side jumps averaged 60.9 ± 6.1 repetitions, and vertical jump height was 55.2 ± 6.1 cm.

Regarding body composition, total body water was 42.8 ± 6.2 L, protein mass was 11.7 ± 1.7 kg, mineral content was 3.9 ± 0.6 kg, fat mass was 7.1 ± 2.7 kg, muscle mass was 55.2 ± 8.0 kg, and fat-free mass was 51.6 ± 19.1 kg. BMI averaged 21.5 ± 1.8, body fat percentage was 10.7 ± 3.5%, skeletal muscle mass was 33.2 ± 4.9 kg, and SMI was 8.3 ± 0.6. Basal metabolic rate was 1631.4 ± 183.2 kcal, and bone mineral content was 3.2 ± 0.5 kg.

Univariate analysis (comparison by overuse injury presence)

Table 2 shows the results of group comparisons based on the presence or absence of overuse injury. Total weekly training time tended to be longer in the overuse injury group (29.1 ± 11.7 h) than in the non-injury group (24.7 ± 7.8 h) (p = 0.053). Daily training time was significantly higher in the overuse injury group (4.3 ± 0.6 h) than in the non-injury group (3.6 ± 1.3 h) (p = 0.034). Mean vertical jump height was significantly lower in the overuse injury group (52.6 ± 4.3 cm) compared to the non-injury group (56.3 ± 6.5 cm) (p = 0.011). SMI tended to be higher in the overuse injury group (8.5 ± 0.6 kg/m²) than in the non-injury group (8.2 ± 0.6 kg/m²) (p = 0.071). No significant differences were observed between the groups in terms of age, height, weight, BMI, years of basketball experience, grip strength, quadriceps thickness, sit-and-reach, side-to-side jumps, body water, protein, minerals, fat mass, muscle mass, fat-free mass, body fat percentage, skeletal muscle mass, basal metabolic rate, or bone mineral content.

**Table 2:** Comparison of demographic characteristics, physical performance, and body composition between athletes with and without overuse injuries ^§^ Quadriceps thickness value adjusted for body weight Between-group comparisons were performed using unpaired t-tests for continuous variables and Fisher’s exact tests for categorical variables. To identify potential variables for subsequent multivariate analyses, a significance threshold of p < 0.1 was used in univariate comparisons. Statistical significance was set at ^*^p < 0.05, and ^†^p < 0.1 was interpreted as a trend toward significance. SD: standard deviation, BMI: body mass index, SMI: skeletal muscle mass index

Variables	Non-overuse injury group (n = 56)		Overuse injury group (n = 24)		p-value
Demographic data; mean ± SD					
Age (years)	16.5	± 0.9	16.5	± 0.9	1.0
Height (cm)	173.6	± 9.7	175.6	± 8.2	0.4
Body weight (kg)	64.7	± 9.2	67.3	± 10.0	0.3
Years of basketball experience (years)	8.7	± 2.6	8.2	± 1.9	0.4
Total weekly training duration (h)	24.7	± 7.8	29.1	± 11.7	0.053^†^
Daily training duration (h)	3.6	± 1.3	4.3	± 0.6	0.034^*^
Weekly training frequency (days/week)	6.6	± 1.0	6.8	± 1.6	0.7
Player positions; n (%)					
Center	5	6.2	2	2.5	0.4
Forward	13	16.1	9	11.1	-
Guard	39	48.2	13	16.1	-
Dietary awareness in relation to training; n (%)					
Do you take care to eat properly before training?					
Yes	15	18.5	7	8.6	0.8
No	42	51.9	17	21.0	-
Do you adjust your diet based on your training volume?					
Yes	14	17.5	11	13.8	0.1
No	42	52.5	13	16.3	-
Physical performance; mean ± SD					
Grip strength (kgf)	41.2	± 7.1	43	± 7.0	0.3
Quadriceps muscle thickness (mm/kg)^§^	41	± 5.4	40.2	± 4.9	0.5
Seated forward bend (cm)	44.3	± 10.1	42.4	± 10.3	0.7
Side-to-side jumps (cm)	61.1	± 5.6	60.2	± 7.3	0.5
Vertical jump (cm)	56.3	± 6.5	52.6	± 4.3	0.011^*^
Body composition parameters; mean ± SD					
Total body water (L)	42.2	± 6.2	44.3	± 5.9	0.2
Protein (kg)	11.5	± 1.7	12.1	± 1.6	0.2
Minerals (kg)	3.8	± 0.6	4	± 0.6	0.2
Body fat mass (kg)	7.2	± 2.5	6.8	± 3.2	0.6
Soft lean mass (kg)	54.4	± 8.0	57.1	± 7.7	0.2
Fat-free mass (kg)	50	± 19.9	55.1	± 16.8	0.3
BMI (kg/m²)	21.4	± 1.8	21.7	± 1.8	0.5
Percent body fat (%)	11	± 3.4	9.9	± 3.9	0.2
Skeletal muscle mass (kg)	32.6	± 4.8	34.5	± 4.8	0.1
Resting energy expenditure (kcal)	1612.8	± 183.9	1675.4	± 177.6	0.2
Bone mineral content (kg)	3.1	± 0.5	3.3	± 0.5	0.2
SMI (kg/m²)	8.2	± 0.6	8.5	± 0.6	0.071^†^

Hierarchical cluster analysis

**Figure 1:**
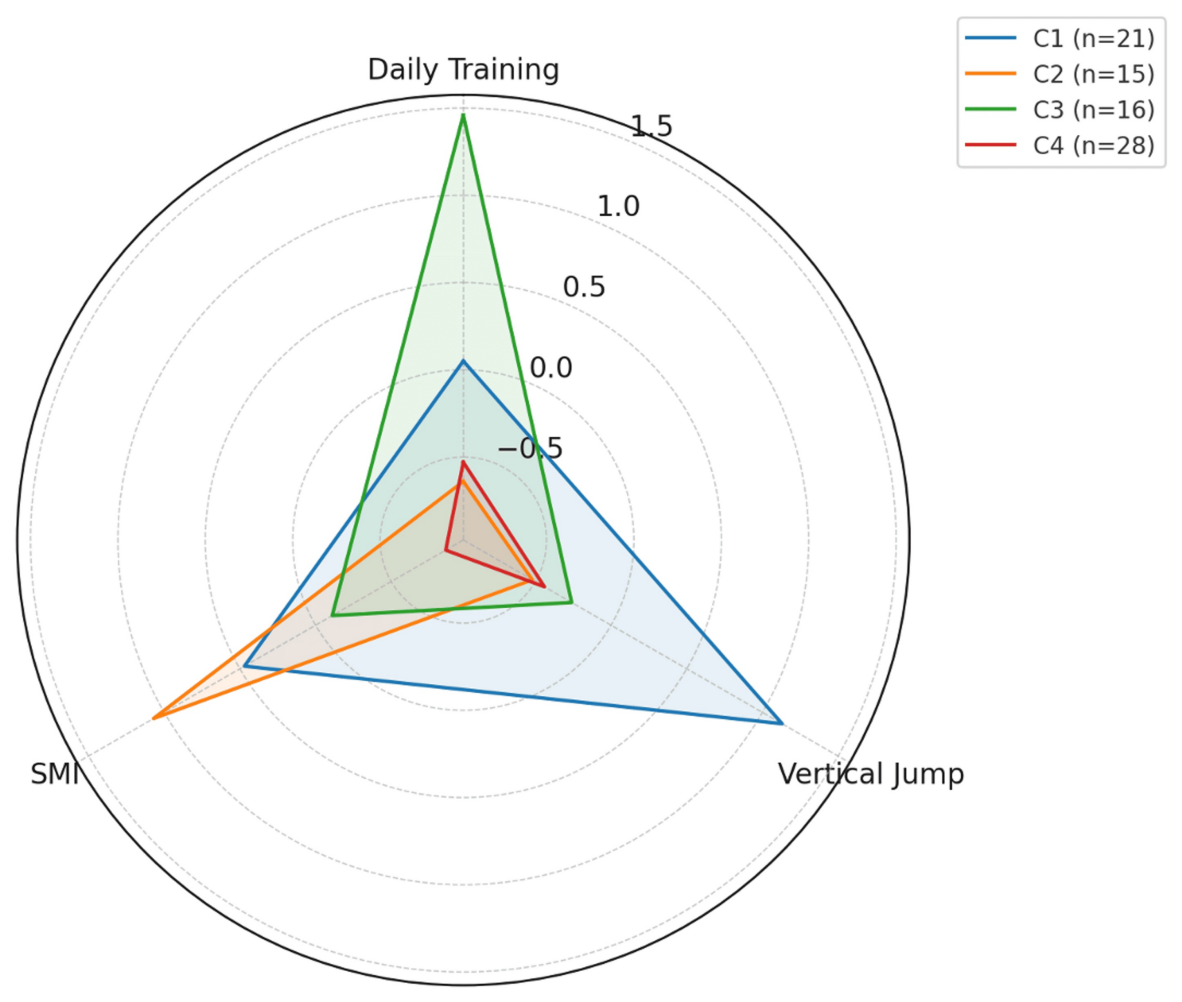
Z-score profiles of each cluster This radar chart visualizes the standardized scores (Z-scores) of three variables, daily training duration, vertical jump height, and skeletal muscle mass index (SMI), for each cluster. Cluster 1 (blue) exhibited the highest vertical jump and moderate values in the other variables, indicating high physical performance. Cluster 2 (orange) had the highest SMI but lower training and jump scores. Cluster 3 (green) had the highest training duration but showed below-average physical function. Cluster 4 (red) demonstrated low scores across all three domains, representing the lowest physical performance cluster. The radar chart was generated using Python (matplotlib), and the source code is available upon request for reproducibility.

Figure 1 illustrates radar charts representing cluster profiles based on the Z-scores of daily training time, vertical jump height, and SMI. The cluster analysis included these three variables, which showed statistical significance or trends in the univariate analysis and were selected for their clinical relevance and representativeness. To avoid redundancy among training load indicators, total weekly training time was excluded from the analysis.

As a result, the 80 participants were classified into the following four clusters: (1) cluster 1 (C1; n = 21), high vertical jump performance (Z = 1.13) and slightly elevated SMI (Z = 0.47), with average training time (Z = 0.05), characterized by high physical performance; (2) cluster 2 (C2; n = 15), highest SMI (Z = 1.07) but low training time (Z = -0.64) and vertical jump (Z = -0.52), characterized by high muscle mass but low physical performance and activity levels; (3) cluster 3 (C3; n = 16), longest training time (Z = 1.46) with below-average vertical jump (Z = -0.26) and SMI (Z = -0.11), characterized by a mismatch between high training load and physical performance; and (4) cluster 4 (C4; n = 28), below-average scores for all indicators-training time (Z = -0.53), vertical jump (Z = -0.44), and SMI (Z = -0.86), characterized by generally low physical function and activity.

The findings suggest that the participants can be divided into four distinct physical characteristic profiles based on training load, muscle mass, and performance.

Results of one-way ANOVA and post hoc comparisons

Table 3 presents the results of one-way ANOVA and effect sizes (η²) for each variable across clusters. Statistically significant differences were observed in all three variables: daily training time (F = 39.82, p < 0.001), SMI (F = 28.73, p < 0.001), and vertical jump height (F = 22.16, p < 0.001). All variables demonstrated large effect sizes: daily training time (η² = 0.61), SMI (η² = 0.53), and vertical jump (η² = 0.47), indicating substantial between-cluster variance.

**Table 3:** Cluster-based comparison of physical characteristics using ANOVA Values are presented as LS mean ± SE. p-values were derived from one-way ANOVA. Effect size η² represents the proportion of variance explained by clusters. Measurement units: daily training duration: hours, SMI: kg/m², vertical jump: cm. Effect size interpretation: 0.01 = small, 0.06 = medium, 0.14 = large (Cohen, 1988 [20]). SMI: skeletal muscle mass index, LS: least squares, SE: standard error, ANOVA: analysis of variance

Variables	Cluster	LS mean ± SE	F-value	p-value	Effect size η^2^
Daily training duration	C1 (n = 21)	3.86 ± 0.17	39.82	< 0.0001	0.61
	C2 (n = 15)	3.00 ± 0.20			
	C3 (n = 16)	5.63 ± 0.20			
	C4 (n = 28)	3.14 ± 0.15			
SMI	C1 (n = 21)	8.55 ± 0.09	28.73	< 0.0001	0.53
	C2 (n = 15)	8.91 ± 0.11			
	C3 (n = 16)	8.21 ± 0.10			
	C4 (n = 28)	7.77 ± 0.08			
Vertical jump	C1 (n = 21)	62.19 ± 1.00	22.16	< 0.0001	0.47
	C2 (n = 15)	52.06 ± 1.18			
	C3 (n = 16)	53.65 ± 1.15			
	C4 (n = 28)	52.55 ± 0.87			

Table 4 summarizes the results of the post hoc comparisons of the key physical characteristics. Cluster 3 had a significantly higher daily training time than all other clusters, with all pairwise comparisons showing an adjusted p < 0.001 and very large effect sizes (d > 1.9).

**Table 4:** Post hoc comparisons of least squares means between clusters for key parameters p-values are Bonferroni-adjusted for six pairwise comparisons per variable. * p < 0.008, ** p < 0.001, n.s.: not significant. Effect size thresholds for interpretation were based on Cohen’s convention: small (d ≥ 0.2), medium (d ≥ 0.5), and large (d ≥ 0.8) (Cohen, 1988 [20]). Mean diff: difference in least squares means between clusters, CI: confidence interval, SMI: skeletal muscle mass index

Variables	Post hoc comparisons	Mean diff	Diff SE	95% CI		Adjusted p		Effect size d
				Lower	Upper			
Daily training duration	C3 vs C2	2.63	0.28	2.06	3.19	< 0.0001	**	2.88
	C3 vs C4	2.48	0.25	1.99	2.98	< 0.0001	**	2.71
	C3 vs C1	1.77	0.26	1.24	2.29	< 0.0001	**	1.98
	C1 vs C2	0.86	0.27	0.32	1.39	0.002	*	1.07
	C1 vs C4	0.71	0.23	0.26	1.17	0.003	*	1.00
	C4 vs C2	0.14	0.25	-0.36	0.65	0.575	n.s.	0.19
SMI	C2 vs C4	1.14	0.13	0.87	1.40	< 0.0001	**	2.71
	C1 vs C4	0.78	0.12	0.54	1.02	< 0.0001	**	1.94
	C2 vs C3	0.69	0.15	0.40	0.99	< 0.0001	**	1.68
	C3 vs C4	0.44	0.13	0.18	0.70	0.003	*	1.07
	C2 vs C1	0.35	0.14	0.08	0.63	0.013	n.s.	0.76
	C1 vs C3	0.34	0.14	0.07	0.61	0.016	n.s.	0.74
Vertical jump	C1 vs C2	10.12	1.56	7.03	13.22	< 0.0001	**	2.94
	C1 vs C4	9.64	1.33	6.99	12.28	< 0.0001	**	2.57
	C1 vs C3	8.53	1.53	5.49	11.58	< 0.0001	**	2.27
	C3 vs C2	1.59	1.65	-1.70	4.88	0.340	n.s.	0.39
	C3 vs C4	1.10	1.44	-1.77	3.97	0.447	n.s.	0.27
	C4 vs C2	0.49	1.47	-2.45	3.42	0.742	n.s.	0.16

Clusters 1 to 3 had significantly higher SMI values than cluster 4. The difference between clusters 2 and 4 was particularly strong (adjusted p < 0.001, d = 2.71). Although the differences between clusters 1 and 2 and between clusters 1 and 3 were not statistically significant after adjustment, both showed moderate effect sizes (d > 0.7).

Regarding vertical jump height, cluster 1 had significantly higher performance than other clusters (all adjusted p < 0.001, d > 2.2), clearly characterizing it as a high-performance group. No significant differences were found between clusters 2 and 4, and the effect sizes were small or negligible.

Association between cluster classification and overuse injury

Table 5 presents the cross-tabulation of overuse injury incidence by cluster classification. Fisher’s exact test indicated a statistically significant association between cluster classification and the occurrence of overuse injury (p = 0.0005). Overuse injury incidence was lowest in cluster 1 (4.8%), whereas clusters 2 and 3 showed high incidence rates of 53.3% and 56.2%, respectively. Cluster 4 had a moderate incidence (21.4 %).

**Table 5:** Cross-tabulation of overuse injury by cluster Cross-tabulation of overuse injury presence by cluster classification. Fisher’s exact test indicated a statistically significant association between cluster classification and overuse injury occurrence (p = 0.0005). Injury rate (%) represents the proportion of overuse injury within each cluster.

Cluster	Overuse injury (-)	Overuse injury (+)	Total	Injury rate (%)
C1	20	1	21	4.8
C2	7	8	15	53.3
C3	7	9	16	56.2
C4	22	6	28	21.4

Logistic regression analysis of overuse injury risk by cluster classification

**Figure 2:**
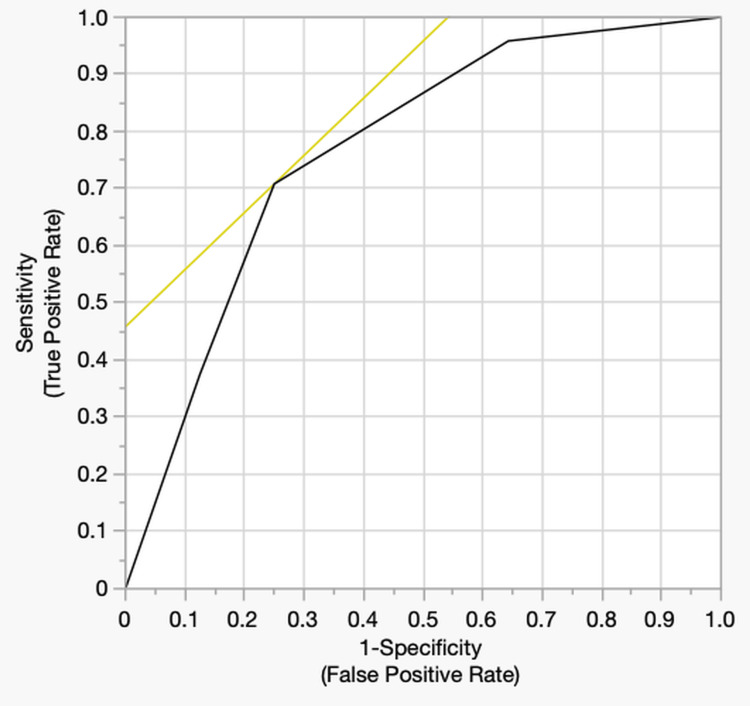
ROC curve of the logistic regression model for predicting overuse injury ROC analysis was performed to evaluate the discriminatory performance of the logistic regression model in predicting the occurrence of overuse injuries. The AUC was 0.77, indicating moderate to good discriminatory accuracy. The yellow line represents the tangent at the selected cutoff point on the ROC curve. ROC: receiver operating characteristic, AUC: area under the curve

Table 6 presents the results of the logistic regression analysis. Compared to the reference group (cluster 1), clusters 2 (OR = 22.9, 95% CI: 2.4-216.9, p = 0.006) and 3 (OR = 25.7, 95% CI: 2.7-241.1, p = 0.005) had significantly higher risks of overuse injury. Although cluster 4 showed an elevated OR of 5.5, the difference was not statistically significant (95% CI: 0.6-49.3, p = 0.131). ROC analysis indicated moderate to good discrimination, with an AUC of 0.77 (Figure 2).

**Table 6:** Logistic regression results: ORs for overuse injury by cluster (cluster 1 as the reference group) ORs and 95% CIs were calculated using logistic regression with cluster 1 as the reference group. Statistical significance was assessed using Wald tests. A Bonferroni correction (adjusted α = 0.008) was applied to account for multiple comparisons. Model fit statistics: −2 log likelihood = 48.86; AIC = 88.33; BIC = 97.32; Nagelkerke’s R² = 0.184 ORs: odds ratios, CIs: confidence intervals

Comparison	OR	95% CI		p-value
		Lower	Upper	
C2 vs C1	22.9	2.4	216.9	0.006
C3 vs C1	25.7	2.7	241.1	0.005
C4 vs C1	5.5	0.6	49.3	0.131

## Discussion

This study identified factors associated with overuse injuries among high school basketball players and used a clustering approach to construct individualized risk profiles. The analysis revealed that daily training duration, vertical jump performance, and SMI were related to the incidence of overuse injuries. Hierarchical cluster analysis revealed four distinct physical profiles based on training volume, muscle mass, and jumping ability. Cluster classification was substantially associated with overuse injury incidence, with clusters 2 and 3 showing a statistically higher risk than cluster 1. Furthermore, ROC analysis of the logistic regression model yielded an AUC of 0.77, indicating moderate to good discrimination.

Previous studies have shown that excessive training loads and insufficient recovery are major risk factors for overuse injury [1,5,6,21], particularly in adolescent athletes whose musculoskeletal immaturity further increases their vulnerability [5]. In our study, the overuse injury group had substantially longer daily training durations, suggesting that load accumulation may have influenced the onset of injury. Reduced vertical jump performance has been linked to an increased risk of overuse injuries. Vertical jump height is a representative indicator of lower limb power and neuromuscular control [22], and poor jump performance may reflect muscular weakness and deficiencies in muscle activation timing and interlimb coordination [23]. Previous studies have reported that young basketball players with large asymmetries in single-leg jump performance tend to have reduced jump and sprint outcomes, as well as higher injury rates [24]. Disruptions in the sequence and timing of muscle activation during jumping may reduce jump height and increase localized stress upon landing, thereby increasing injury risk [25-27]. Thus, diminished jump performance likely reflects immature neuromuscular control, contributing to the development of overuse injuries.

A key finding of this study was the classification of the four distinct risk profiles using a clustering method based on physical characteristics and training load. These profiles provide a structural understanding of injury risk and may support the development of individualized prevention strategies. Cluster 1, the "high physical function group," demonstrated the highest vertical jump performance, above-average SMI, and average training time. With the lowest overuse injury rate (4.8%), this group represents an optimal balance between physical function and training load management for injury prevention. Cluster 2, the "high SMI, low training group," showed high muscle mass but low jump performance and training time. Despite low training exposure, this group had a high incidence of injury (53.3%), suggesting that muscle mass alone does not confer protection. This mismatch between structural (muscle mass) and functional (output and coordination) capacities may be a key risk factor for overuse injuries in adolescent athletes. Additionally, poor movement patterns and underdeveloped motor control may contribute to injury, even with low training loads.

Cluster 3, the "high training, low function group," displayed the highest training duration but subaverage physical performance, with the highest injury incidence (56.2%). This supports Gabbett's theory on the mismatch between fitness and training load [7], indicating that an excessive load on underprepared athletes increases injury risk [28]. Early screening and training modifications are essential in this group, making it a critical target for immediate intervention. Cluster 4, the "low function, low training group," had a moderate injury rate (21.4%). Although this group may be at latent risk due to poor physical function, a low training load may suppress overt injuries. However, future increases in training load could elevate the risk of injury, highlighting the importance of early functional enhancement in this group.

These risk profiles provide a multidimensional understanding of injury mechanisms in individual athletes, offering practical value for screening and targeted interventions. Logistic regression results confirmed the statistical association between cluster classification and injury incidence, and ROC analysis (AUC = 0.77) further demonstrated the model's utility for field-based screening. A major strength of this study is its integrative evaluation of body composition, physical function, and training load factors, which are often analyzed separately. This study provides a foundation for refined injury prevention by visualizing complex risk structures through clustering. Additionally, the selected variables were easily measurable in field settings, thereby enhancing the model's applicability and feasibility. These findings underscore the importance of profiling based on interactions between multiple physical characteristics and sports-specific demands.

This study has some limitations worth noting. First, as an observational study, causal relationships could not be confirmed, and intervention studies are needed to validate the effectiveness of these profiles. Second, the sample was limited to male high school basketball players from a single elite-level team; therefore, generalizations to other sexes, sports, and competitive contexts should be made with caution. Third, psychological and social factors (such as stress, self-efficacy, and sleep) were not assessed; future research should incorporate these variables for a more comprehensive risk evaluation. Fourth, because overuse injury data were collected using self-reported questionnaires, there may be a risk of information or recall bias. Although participants were asked to respond accurately, some degree of under- or overreporting cannot be entirely ruled out. Finally, although trained examiners followed standardized procedures to ensure consistency, and players with acute injuries or pain that interfered with testing were excluded, minor variations in measurement or participant effort may still have occurred. Building on the findings of this study, future research should test individualized prevention programs tailored to these profiles and examine their applicability across various sports and demographics. Developing more inclusive injury risk models that incorporate psychosocial elements should also be considered.

## Conclusions

This study provides practical insights by systematically classifying and visualizing overuse injury risks through an integrative analysis of body composition, physical performance, and training load. By employing field-accessible indicators, the proposed approach enhances the feasibility of early screening in real-world settings. These findings underscore the importance of maintaining an appropriate balance between physical capacity and training exposure to prevent overuse injuries. Moreover, the application of cluster-based profiling demonstrates strong potential for early risk identification and the development of individualized, data-driven intervention strategies for athletes.
